# Occupational exposure to asbestos and mortality among asbestos removal workers: a Poisson regression analysis

**DOI:** 10.1038/sj.bjc.6604798

**Published:** 2008-11-25

**Authors:** G Frost, A-H Harding, A Darnton, D McElvenny, D Morgan

**Correction to**: *British Journal of Cancer* (2008) **99**, 822–829, doi:10.1038/sj.bjc.6604564

Owing to an error during typesetting, there were some mistakes introduced into the layouts of Tables 2 and 3 in the above paper when first published in the *British Journal of Cancer* in September 2008. The presentation of the tables as such may have led to misinterpretation of data; indeed, some data were presented incorrectly in Table 3.

The tables are now reproduced, as the author intended, on the following pages.

The publishers would like to apologise for these errors.

## Figures and Tables

**Table 2:** Standardised mortality ratios (SMR) for all asbestos removal workers in the survey and for those included in the analysis (1971–2005)

	**All removal workers**		**Analysed workers**	
**Cause of death**	**Deaths**	**SMR (95% CI)**	**Deaths**	**SMR (95% CI)**
All causes	3165	122.9^**^	985	111.3^**^
		(118.7–127.3)		(104.4–118.4)
All MN	1274	173.1^**^	384	169.2^**^
		(163.7–182.9)		(152.7–187.0)
MN of lip, oral cavity and pharynx	19	94.0	6	83.7
		(56.6–146.9)		(30.7–182.2)
MN of oesophagus	42	88.2	16	104.2
		(63.5–119.2)		(59.6–169.2)
MN of stomach	49	133.7	18	176.4^*^
		(98.9–176.8)		(104.5–278.8)
MN of colon	62	127.9	17	119.2
		(98.1–163.9)		(69.4–190.9)
MN of rectum	43	169.0^**^	15	207.1^*^
		(122.3–227.6)		(115.9–341.5)
MN of liver (primary)	20	126.6	9	167.8
		(77.3–195.5)		(76.7–318.6)
MN of larynx	17	212.3^**^	8	322.9^**^
		(123.6–339.8)		(139.4–636.1)
MN trachea, bronchus and lung	393	200.5^**^	115	215.6^**^
		(181.2–221.4)		(178.0–258.7)
MN of peritoneum	38	4568.9^**^	12	4367.8^**^
		(3233.2–6271.2)		(2257.0–7629.8)
MN of pleura	35	1149.3^**^	10	1332.8^**^
		(800.5–1598.4)		(639.1–2451.1)
Mesothelioma^a^	69	808.2^**^	23	808.2^**^
		(628.8–1022.8)		(512.3–1212.7)
MN of ovary	0	—	0	—
		(0.0–533.0)		(0.0–7092.9)
MN of kidney	29	160.1^*^	13	229.0^*^
		(107.2–229.9)		(121.9–391.6)
MN of bladder	18	94.2	3	61.6
		(55.8–148.8)		(12.7–180.2)
MN of lymphatic and haematopoietic tissue	80	103.9	24	86.2
		(82.4–129.3)		(55.2–128.2)
Circulatory disease	981	114.0^**^	258	102.0
		(107.0–121.4)		(89.9–115.2)
Ischaemic heart disease	665	113.3^**^	171	102.4
		(104.9–122.3)		(87.7–119.0)
Cerebrovascular disease	147	125.0^*^	41	118.3
		(105.6–146.9)		(84.9–160.5)
Respiratory disease	222	128.0^**^	44	88.8
		(111.7–146.0)		(64.5–119.2)
Asbestosis^b^	22	5753.4^**^	3	3849.6^**^
		(3605.6–8710.7)		(794.3–11249.8)

CI=confidence interval.

^*^Significant at *P*⩽0.05; ^**^significant at *P*⩽0.01.

a ICD-10, after 2001.

b Asbestosis determined by underlying cause of death.

**Table 3:** Relative risks (RRs) of mortality for stripping/removal workers, using Poisson regression analyses

	**All causes**			**All MN**			**MN trachea, bronchus and lung**		
	**Cases**	**RR^a^ (95% CI)**	**LR test (d.f.)**	**Cases**	**RR^a^ (95% CI)**	**LR test (d.f.)**	**Cases**	**RR^a^ (95% CI)**	**LR test (d.f.)**
*Main stripping method*			4.03 (2)			2.35 (2)			2.71 (2)
Wet^c^	397	1.0		141	1.0		48	1.0	
Dry	409	1.1 (0.9–1.2)		169	1.2 (0.9–1.5)		39	0.8 (0.5–1.2)	
Both	48	0.8 (0.6–1.1)		21	1.0 (0.6–1.6)		13	1.3 (0.7–2.7)	
*Main respirator type*			2.82 (6)			10.20 (6)			6.28 (6)
Positive pressure mask^c^	664	1.0		245	1.0		70	1.0	
Airstream helmet	4	—		1	—		1	—	
Full face unpowered mask	127	1.0 (0.8–1.2)		55	1.1 (0.8–1.5)		16	1.1 (0.6–1.8)	
Half face mask	18	0.8 (0.5–1.3)		4	—		1	—	
Minimal	14	0.7 (0.4–1.3)		5	—		2	—	
None	2	—		2	—		1	—	
Mixed	51	1.1 (0.8–1.5)		25	1.4 (0.9–2.2)		11	1.7 (0.9–3.5)	
*Weekly hours spent stripping*			14.97 (4)^**^			1.00 (4)			0.87 (4)
<10^c^	483	1.0		222	1.0		66	1.0	
10−	74	1.0 (0.8–1.2)		32	1.0 (0.7–1.5)		8	0.9 (0.4–1.8)	
20−	125	1.1 (0.9–1.4)		48	1.1 (0.8–1.5)		12	1.0 (0.5–1.8)	
30−	124	1.3 (1.0–1.5)^*^		33	0.9 (0.6–1.3)		9	0.9 (0.4–1.7)	
40+	163	1.4 (1.2–1.7)^**^		44	1.0 (0.7–1.4)		15	1.2 (0.7–2.1)	
*Smoking status*			112.42 (2)^**^			41.07 (2)^**^			67.66 (2)^**^
Never smokers^c^	118	1.0		38	1.0		1	1.0	
Current smokers	622	2.5 (2.0–3.0)^**^		216	2.8 (1.9–3.9)^**^		86	43.0 (6.0–305.8)^**^	
Former smokers	225	1.5 (1.2–1.9)^**^		125	2.2 (1.5–3.1)^**^		26	16.0 (2.2–118.4)^**^	
*Age at first exposure* (*years)*			14.80 (4)^**^			13.41 (4)^**^			3.22 (4)
<20^c^	184	1.0		91	1.0		21	1.0	
20−	278	1.1 (0.9–1.3)		81	0.9 (0.7–1.2)		18	1.0 (0.5–1.8)	
30−	193	0.9 (0.8–1.1)		70	0.8 (0.6–1.1)		23	1.2 (0.7–2.1)	
40−	211	1.1 (0.9–1.3)		84	0.7 (0.5–1.0)^*^		33	1.2 (0.7–2.0)	
50+	119	0.7 (0.6–0.9)^**^		58	0.6 (0.4–0.8)^**^		20	0.7 (0.4–1.3)	
*Length of time in the survey*			0.01 (1)			14.81 (1)^**^			5.13 (1)^*^
Short term^c^	419	1.0		116	1.0		33	1.0	
Long term	599	1.0 (0.9–1.1)		268	1.5 (1.2–1.9)^**^		82	1.6 (1.1–2.4)^*^	
*Length of exposure (years)*			16.29 (4)^**^			18.61 (4)^**^			6.88 (4)
<10^c^	553	1.0		166	1.0		49	1.0	
10−	161	1.0 (0.8–1.2)		60	1.1 (0.8–1.5)		26	1.6 (1.0–2.5)	
20−	88	0.8 (0.7–1.0)		45	1.1 (0.8–1.5)		11	0.8 (0.4–1.6)	
30−	94	1.0 (0.8–1.2)		61	1.4 (1.1–1.9)^*^		14	1.0 (0.5–1.8)	
40+	89	1.5 (1.2–2.0)^**^		52	2.1 (1.5–2.9)^**^		15	1.7 (0.9–3.2)	
*Time of first exposure*			0.00 (1)			4.02 (1)^*^			1.43 (1)
Pre-ALR^c^	430	1.0		217	1.0		66	1.0	
Post-ALR	555	1.0 (0.9–1.1)		167	0.8 (0.7–1.0)^*^		49	0.8 (0.5–1.2)	
	**Mesothelioma^b^**			**Circulatory disease**			**Ischaemic heart disease**		
	**Cases**	**RR^a^ (95% CI)**	**LR test (d.f.)**	**Cases**	**RR^a^ (95% CI)**	**LR test (d.f.)**	**Cases**	**RR^a^ (95% CI)**	**LR test (d.f.)**
*Main stripping method*			3.25 (2)			6.81 (2)^*^			8.61 (2)^*^
Wet^c^	12	1.0		109	1.0		77	1.0	
Dry	10	0.9 (0.4–2.1)		107	1.0 (0.7–1.3)		66	0.9 (0.6–1.2)	
Both	0	—		7	0.4 (0.2–0.9)^*^		3	—	
*Main respirator type*			10.14 (6)			1.54 (6)			2.24 (6)
Positive pressure mask^c^	20	1.0		172	1.0		110	1.0	
Airstream helmet	0	—		1	—		1	—	
Full face unpowered mask	1	—		34	1.0 (0.7–1.4)		24	1.1 (0.7–1.7)	
Half face mask	0	—		7	1.1 (0.5–2.5)		5	—	
Minimal	0	—		4	—		2	—	
None	1	—		0	—		0	—	
Mixed	1	—		12	1.0 (0.5–1.7)		9	1.2 (0.6–2.3)	
*Weekly hours spent stripping*			2.29 (1)			12.34 (4)^*^			11.42 (4)^*^
<10^c^	17	1.0		133	1.0		86	1.0	
10−	6	0.5 (0.2–1.3)		16	0.8 (0.5–1.4)		12	1.0 (0.5–1.8)	
20−	—			28	1.1 (0.7–1.6)		16	0.9 (0.6–1.6)	
30−	—			32	1.4 (0.9–2.1)		24	1.6 (1.0–2.6)^*^	
40+	—			47	1.7 (1.2–2.4)^**^		32	1.9 (1.2–2.8)^**^	
*Smoking status*			2.27 (2)			33.41 (2)^**^			35.14 (2)^**^
Never smokers^c^	5	1.0		38	1.0		19	1.0	
Current smokers	7	0.7 (0.2–2.3)		161	2.1 (1.4–2.9)^**^		114	2.9 (1.8–4.7)^**^	
Former smokers	11	1.5 (0.5–4.4)		53	0.9 (0.6–1.4)		34	1.2 (0.7–2.1)	
*Age at first exposure* *(years)*			22.12 (1)^**^			12.38 (4)^*^			12.71 (4)^*^
<20^c^	14	1.0		44	1.0		33	1.0	
20−	9	0.1 (0.1–0.3)^**^		41	0.8 (0.5–1.2)		19	0.5 (0.3–0.9)^*^	
30−	—			51	1.0 (0.7–1.6)		34	0.9 (0.6–1.5)	
40−	—			78	1.5 (1.0–2.2)^*^		54	1.3 (0.9–2.1)	
50+	—			44	0.9 (0.6–1.4)		31	0.9 (0.5–1.5)	
*Length of time in the survey*			8.23 (1)^**^			0.00 (1)			0.60 (1)
Short term^c^	3	1.0		102	1.0		72	1.0	
Long term	20	4.5 (1.3–16.0)^*^		156	1.0 (0.8–1.3)		99	0.9 (0.6–1.2)	
*Length of exposure (years)*			18.59 (1)^**^			7.19 (4)			10.39 (4)^*^
<10^c^	—			141	1.0		92	1.0	
10−	—			48	1.0 (0.7–1.4)		36	1.2 (0.8–1.7)	
20−	9^d^	1.0		27	0.8 (0.5–1.2)		14	0.6 (0.3–1.0)	
30−	14	7.3 (2.5–21.6)^**^		20	0.6 (0.4–0.9)^*^		13	0.6 (0.3–1.0)^*^	
40+	—			22	0.9 (0.6–1.5)		16	1.1 (0.6–1.9)	
*Time of first exposure*			13.15 (1)^**^			0.46 (1)			0.84 (1)
Pre-ALR^c^	19	1.0		123	1.0		81	1.0	
Post-ALR	4	—		135	1.1 (0.8–1.4)		90	1.2 (0.8–1.6)	
	**Cerebrovascular disease**			**Respiratory disease**					
	**Cases**	**RR^a^ (95% CI)**	**LR test (d.f.)**	**Cases**	**RR^a^ (95% CI)**	**LR test (d.f.)**			
*Main stripping method*			0.62 (2)			2.46 (2)			
Wet^c^	18	1.0		23	1.0				
Dry	14	0.8 (0.4–1.5)		13	0.6 (0.3–1.2)				
Both	2	—		4	—				
*Main respirator type*			7.12 (6)			4.02 (6)			
Positive pressure mask^c^	31	1.0		33	1.0				
Airstream helmet	0	—		0	—				
Full face unpowered mask	4	—		4	—				
Half face mask	0	—		0	—				
Minimal	1	—		1	—				
None	0	—		0	—				
Mixed	0	—		3	—				
*Weekly hours spent stripping*			0.68 (2)			3.46 (3)			
<10^c^	21	1.0		21	1.0				
10−	10	1.4 (0.6–2.9)		8	1.1 (0.5–2.5)				
30−	10	1.2 (0.6–2.7)		7	2.0 (0.8–4.6)				
40+	—			8	1.9 (0.9–4.1)				
*Smoking status*			5.96 (2)			14.18 (2)^**^			
Never smokers^c^	12	1.0		2	1.0				
Current smokers	22	0.9 (0.4–1.8)		30	7.4 (1.8–30.6)^**^				
Former smokers	6	0.3 (0.1–0.9)^*^		11	3.8 (0.8–17.2)				
*Age at first exposure* (*years)*			3.04 (4)			4.35 (3)			
<20^c^	6	1.0		6	1.0				
20−	8	1.1 (0.4–3.2)		12	1.7 (0.7–4.8)				
30−	8	1.5 (0.5–4.7)		12	2.1 (0.8–5.3)				
40−	12	2.2 (0.8–6.6)		14	1.0 (0.4–2.6)				
50+	7	1.3 (0.4–4.4)		—					
*Length of time in the survey*			0.45 (1)			0.46 (1)			
Short term^c^	15	1.0		20	1.0				
Long term	26	1.2 (0.7–2.3)		24	0.8 (0.5–1.5)				
*Length of exposure (years)*			0.94 (2)			7.14 (3)			
<10^c^	24	1.0		25	1.0				
10−	6	0.8 (0.3–1.9)		6	0.4 (0.2–1.1)				
20−	11	0.7 (0.3–1.5)		—					
30−	—			6	1.1 (0.4–2.7)				
40+	—			7	1.9 (0.8–4.4)				
*Time of first exposure*			1.16 (1)			0.01 (1)			
Pre-ALR^c^	18	1.0		22	1.0				
Post-ALR	23	1.4 (0.7–2.8)		22	1.0 (0.5–1.8)				

CI=confidence interval; d.f.=degrees of freedom; LR test=likelihood ratio test of goodness-of-fit.

^*^significant at *P*⩽0.05; ^**^significant at *P*⩽0.01.

a Relative risk adjusted with Poisson regression for age, calendar period and sex.

b ICD-10, after 2001.

c Reference category.

d Categories: <30, 30+.

